# Psychometric evaluation of the Urgency NRS as a new patient-reported outcome measure for patients with ulcerative colitis

**DOI:** 10.1186/s41687-022-00522-2

**Published:** 2022-11-05

**Authors:** Marla C. Dubinsky, Mingyang Shan, Laure Delbecque, Trevor Lissoos, Theresa Hunter, Gale Harding, Larissa Stassek, David Andrae, James D. Lewis

**Affiliations:** 1 Icahn School of Medicine, Mount Sinai, NY USA; 2 Eli Lilly and Company, Indianapolis, IN USA; 3 Evidera, Bethesda, MD USA; 4 Division of Gastroenterology and Hepatology, Pereleman School of Medicine, University of Pennsylvania, Philadelphia, PA USA

**Keywords:** Bowel urgency, Colitis, Ulcerative, Minimal Clinically Important Difference, Patient Reported Outcome Measures, Psychometric evaluation, Remission induction, Urgency NRS, Validation

## Abstract

**Background:**

The Urgency Numeric Rating Scale (NRS) was developed as a content-valid single-item patient-reported outcome measure to assess severity of bowel urgency. Here, we evaluated the psychometric properties of the Urgency NRS.

**Methods:**

Data were from a multicenter, randomized, placebo-controlled phase 3 trial in adults with moderately to severely active ulcerative colitis (NCT03518086). Patients completed the Urgency NRS using a daily electronic diary, from which weekly average Urgency NRS scores were calculated. Test–retest reliability, known-groups validity, construct validity, responsiveness, and score interpretation were assessed using the modified Mayo score, Inflammatory Bowel Disease Questionnaire (IBDQ), Patient Global Rating of Severity (PGRS), Patient Global Rating of Change (PGRC), and Geboes score.

**Results:**

The study sample comprised 1,162 participants (40.2% female). Mean Urgency NRS score was higher (worse) at baseline than at week 12 (6.2 vs. 3.7). Test–retest reliability was strong, with intra-class correlation coefficients of 0.76–0.89. Baseline least-square mean Urgency NRS score was higher for participants with a PGRS score greater than the median (worse symptoms) than for those with a PGRS score less than or equal to the median (7.5 vs. 5.4; *p* < 0.0001), indicating good known-groups validity. Urgency NRS score was moderately correlated with IBDQ total and domain scores, PGRS, PGRC, and modified Mayo stool frequency, establishing its convergent validity. Correlations were weak for Geboes score and weak to moderate for modified Mayo endoscopic subscore and modified Mayo rectal bleeding, indicating that the Urgency NRS also had discriminant validity. Patients achieving clinical remission, clinical response, IBDQ remission, and PGRS score improvement showed significantly greater improvement on the Urgency NRS (*p* < 0.0001 for all), demonstrating responsiveness to change. A ≥ 3-point improvement in Urgency NRS score represented a meaningful improvement in bowel urgency and an Urgency NRS score of ≤ 1 point represented a bowel urgency remission threshold that was closely associated with clinical, endoscopic, and histologic remission.

**Conclusions:**

The Urgency NRS is a valid and reliable patient-reported outcome measure that is suitable for evaluating treatment benefits in clinical trials in patients with moderately to severely active ulcerative colitis.

**Supplementary Information:**

The online version contains supplementary material available at 10.1186/s41687-022-00522-2.

## Background

Ulcerative colitis (UC) is a chronic disease of unknown etiology that is characterized by inflammation of the colon and rectum. Common symptoms of UC include blood in the stool, diarrhea, and bowel urgency [1]. Bowel urgency is highly bothersome, and reducing its severity has been identified as a key factor influencing patients’ treatment decisions [2]. Clinical guidelines also identify bowel urgency as an important disease-related symptom signifying the severity of disease activity and recommend control of urgency as part of the management of UC [3]. However, bowel urgency has not been specifically assessed in clinical trials of UC treatments. Moreover, until recently, no validated patient-reported outcome (PRO) measures were available for specifically assessing changes in its severity resulting from treatment of UC.

To address this, we developed a new PRO measure, the Urgency Numeric Rating Scale (NRS), through a targeted literature review, concept elicitation interviews with UC patients, and expert input [4, 5]. Content validity of the Urgency NRS was previously established through cognitive interviews with adult UC patients [4, 5]. The aims of the present study were to evaluate the reliability, validity, and responsiveness of the instrument in a clinical trial setting; to identify a score change representing clinical meaningful improvement in bowel urgency; and to identify a bowel urgency severity threshold associated with inactive disease or remission.

## Methods

The data used in this study were from LUCENT-1, a phase 3 randomized, double-blind, parallel-arm trial of the safety and efficacy of mirikizumab for UC induction treatment in adults with moderately to severely active UC (NCT03518086) [6]. Participants were randomly assigned 3:1 to receive an intravenous infusion of mirikizumab 300 mg or placebo at weeks 0, 4, and 8 during a 12-week treatment period. The primary outcome in LUCENT-1 was the percentage of participants in clinical remission of UC at week 12 based on a modified Mayo score (MMS) [7].

### Participants

Participants were adults (aged 18–80 years) diagnosed with UC at least 3 months previously and with lack of response, loss of response, or intolerance to one of the following: corticosteroids, azathioprine, 6-mercaptopurine, infliximab, adalimumab, golimumab, vedolizumab, or tofacitinib. At baseline, their UC extended beyond the rectum and was moderately to severely active, as defined by an MMS total score of 4 to 9 and an endoscopic subscore ≥ 2. Patients with Crohn’s disease, unclassified inflammatory bowel disease, or UC not extending beyond the rectum, or who had undergone colectomy, were excluded. All participants provided written informed consent.

### Patient-reported outcomes

#### Urgency NRS

**Fig. 1:**
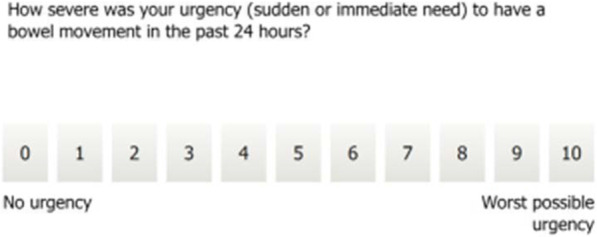
The urgency NRS

The Urgency NRS [5] is a single-item measure of bowel urgency severity in the previous 24 h (Fig. 1). Bowel urgency is scored on an 11-point NRS ranging from 0 (no urgency) to 10 (worst possible urgency). Patients completed the Urgency NRS as part of a daily electronic diary (eDiary). Weekly average scores for the Urgency NRS were subsequently calculated (to the nearest whole number) for 7-day periods. A weekly score was considered missing if fewer than 4 days of scores were available in a given week.

#### Inflammatory Bowel Disease Questionnaire

The Inflammatory Bowel Disease Questionnaire (IBDQ) [8, 9] is a 32-item PRO instrument comprising four domains: bowel symptoms, systemic symptoms, emotional functioning, and social functioning. Each item is scored on a 7-point Likert scale ranging from 1 (“a very severe problem”) to 7 (“not a problem at all”). The total score ranges from 32 to 224, with a higher score indicating better quality of life. The IBDQ was completed at screening, baseline, and week 12. IBDQ remission was defined as an IBDQ total score ≥ 170 [10].

#### Patient Global Rating of Severity

The Patient Global Rating of Severity (PGRS) is a single-item PRO measure for assessing overall disease symptom severity over the previous 24 h on a 6-point scale from 1 (“none”) to 6 (“very severe”). The PGRS was completed as part of the daily eDiary to assess UC severity. A weekly average score was calculated in the same way as for the Urgency NRS. At week 12, a ≥ 2-point improvement on the PGRS from baseline was prespecified as a large and meaningful improvement in symptom severity, and a PGRS score of 1 or 2 was considered indicative of UC symptom remission or minimal symptom severity [11–13].

#### Patient Global Rating of Change

The Patient Global Rating of Change (PGRC) is a single-item PRO measure of change in overall symptoms since starting a new medicine. Responses are graded on a 7-point scale from 1 (“very much better”) to 7 (“very much worse”). The PGRC was completed at weeks 4, 8, and 12 to assess change in UC severity. A PGRC score of 1 (“very much better”) or 2 (“much better”) at week 12 was prespecified as a large and meaningful improvement in symptom severity [11–13].

### Disease activity measures

#### Modified Mayo score

The MMS comprises three subscores: stool frequency, rectal bleeding, and the endoscopic subscore. Each of the subscores is scored on a scale of 0 to 3. The MMS total score (range 0 to 9) is derived by summing the scores for the three subscales. In the present study, patients recorded stool frequency and rectal bleeding subscores daily as part of the eDiary. Weekly scores for the stool frequency and rectal bleeding subscales were calculated as the average of the three most recent non-missing daily scores in a 7-day scoring period. The weekly rectal bleeding and stool frequency subscores were considered missing if fewer than 3 days of diary data were available. The endoscopic subscore was scored based on analysis of biopsy samples collected at baseline and week 12 by both the site endoscopist and the blinded central reader using a predefined algorithm. Clinical remission based on Mayo subscores was defined as a stool frequency subscore of 0, or 1 with a ≥ 1-point decrease from baseline; a rectal bleeding subscore of 0; and an endoscopic subscore of 0 or 1 (excluding friability) [14]. Clinical response was defined as a decrease in MMS total score from baseline of ≥ 2 points and ≥ 30%; and a rectal bleeding subscore of 0 or 1, or that had decreased by ≥ 1 point from baseline [14]. An endoscopic subscore of 0 or 1 (excluding friability) indicated endoscopic remission [14].

#### Histology

Participants underwent lower endoscopy at baseline and week 12. Biopsy samples were collected during endoscopic procedures, provided it was safe to collect them, and were analyzed histopathologically by blinded central readers using the Geboes scoring system [15]. The Geboes scoring system assigns values to seven histologic features: 0 structural (architectural change), 1 chronic inflammatory infiltrate, 2a lamina propria eosinophils, 2b lamina propria neutrophils, 3 neutrophils in epithelium, 4 crypt destruction, and 5 erosion or ulceration. Erosion or ulceration is scored on 5 levels; other features are scored on 4 levels. Histologic remission was defined as a Geboes histologic score of 2b (absence of neutrophils in the epithelium and lamina propria; no crypt destruction, erosion, or ulceration) [16].

### Statistical analysis

#### Psychometric analysis of the Urgency NRS

The psychometric properties of the Urgency NRS were analyzed using established methods [17] among patients with moderately to severely active UC using data from LUCENT-1. Trial participants from the modified intent-to-treat population, which included all randomized patients who received at least one dose of study drug, were pooled across treatment arms. The statistical analyses were conducted as specified in a psychometric validation analysis plan using SAS® version 9.4 or later (SAS Institute, Cary, NC). All analyses are as observed on the weekly average Urgency NRS score and other assessments unless otherwise specified.

Descriptive statistics were calculated for participant demographics and for average weekly Urgency NRS scores. In addition, the distributions of the daily Urgency NRS scores in the 1-week periods prior to the baseline and week 12 clinical visits were also evaluated. Possible floor and ceiling effects for Urgency NRS scores were evaluated to ensure that participants did not disproportionately report the lowest or highest possible score (0 or 10) at baseline or week 12.

Shrout and Fleiss intraclass correlation coefficients (ICC(2,1)) were estimated to evaluate the test–retest reliability among ‘stable’ patients [18, 19]. Two groups of stable participants were defined: those with no change in PGRS score between screening and baseline and those registering “no change” on the PGRC at week 4 compared to baseline. An ICC ≥ 0.70 was considered evidence of acceptable test–retest reliability [20]. ICC(2,1) was calculated using two-way random effects models with subject and time as random effects [21, 22]. Modified large sample confidence intervals were constructed for the ICC(2,1) according to Cappelleri and Ting (2003) [23].

Known-groups validity of the Urgency NRS was evaluated at baseline by comparing the distribution of the Urgency NRS between patients who had a PGRS $\le$ median compared to those with a PGRS score > median at baseline. In addition, known groups validity of the Urgency NRS was evaluated at week 12 according to the following groups at week 12: PGRS score ≤ median or > median, clinical remission status, and clinical response status. Least-square (LS) mean scores on the Urgency NRS at baseline or week 12 were compared between known groups using analysis of variance models that included Urgency NRS score as the dependent variable and group as the independent variable. Cohen’s d was calculated as a standardized measure of mean difference between known groups at baseline and week 12. It was hypothesized that patients with more severe UC symptoms (higher PGRS scores at baseline and week 12, clinical non-responders at week 12, and clinical non-remitters at week 12) would have higher Urgency NRS scores.

Convergent validity was assessed by calculating Spearman correlation coefficients at baseline and week 12 between the Urgency NRS and IBDQ total and domain scores, PGRS, PGRC (week 12 only), Mayo rectal bleeding subscore, and Mayo stool frequency subscore. Discriminant validity was assessed by calculating Spearman correlations for the Urgency NRS with Geboes score and Mayo endoscopic subscore as objective measures at baseline and week 12. Cohen’s conventions were used to interpret the magnitude of the correlations: a correlation < 0.1 was considered negligible, between 0.1 and 0.3 was weak, between 0.3 to 0.5 was moderate, and > 0.5 was considered strong [24]. It was hypothesized that Urgency NRS scores would have moderate to strong correlations with IBDQ total score, PGRS, PGRC, Mayo stool frequency subscore, and Mayo rectal bleeding subscore and weak correlations with Geboes score and Mayo endoscopic subscore.

Responsiveness was evaluated by comparing mean changes in Urgency NRS scores from baseline to week 12 between groups of patients with and without meaningful improvements at week 12 according to clinical remission and clinical response (based on MMS total score and Mayo subscores), IBDQ remission, median PGRS score, uncollapsed PGRS score changes (4-point decrease through 1-point increase), and uncollapsed PGRC categories (“very much better” through “very much worse”). Effect sizes were calculated as a standardized measure of improvement on the Urgency NRS between groups at week 12 by dividing the difference in change from baseline between groups by the pooled standard deviation at baseline. One-way analysis of covariance models were used to compare the LS mean change from baseline between groups, with change in Urgency NRS score as the dependent variable, and baseline Urgency NRS score and the meaningful improvement group as independent variables. Scheffe’s correction was used for pairwise comparisons.

#### Urgency NRS score interpretation

Anchor-based analyses were conducted to identify a threshold for meaningful, within-patient improvement in Urgency NRS score, with PGRC, PGRS, and clinical remission serving as anchor variables [23, 25–27]. Spearman correlations were calculated between change from baseline to Week 12 on the Urgency NRS with change from baseline to Week 12 on the PGRS, MMS, and the Week 12 PGRC to assess the appropriateness of the anchor variables (correlation ≥ 0.3 was required). A large and meaningful improvement in symptom severity at week 12 was defined as a PGRS improvement of ≥ 2 points and a PGRC score of 1 (“very much better”) or 2 (“much better”). Sensitivity, specificity, positive predictive value, negative predictive value, and Youden’s index (YI) (sensitivity + specificity − 1) [28] were calculated for each possible Urgency NRS improvement threshold to correctly act as a surrogate for meaningful improvement compared to other levels of improvement, no change, or worsening according to the anchor variable. In addition, area under the receiver operating characteristic curve (AUROC) was calculated from a logistic regression with the anchor variable as the dependent variable and urgency improvement status as defined by the change from baseline threshold on the Urgency NRS as the independent variable [29]. The Urgency NRS score change that maximized YI and AUROC were considered candidate thresholds for meaningful within-patient change in Urgency NRS score.

Resolution or near resolution of symptoms is an important treatment goal in UC. Anchor-based analyses were performed to explore the levels of urgency severity that are most associated with patients being in remission or inactive disease and reflect bowel urgency remission at week 12. Clinical remission, endoscopic remission, histologic remission, and a PGRS score of 1 or 2 were used as binary remission anchor variables reflecting being or not being in a state of remission or inactive disease. Sensitivity, specificity, positive predictive value, negative predictive value, and YI were calculated for a sequence of thresholds on the Urgency NRS against the anchor variables as the ground truth. AUROC was calculated from a logistic regression with the anchor variable as the dependent variable and urgency remission status as defined by the Urgency NRS threshold at week 12 as the independent variable. Urgency NRS scores with the largest Youden’s index and AUROC values were considered as candidate thresholds, below which patients were considered to have bowel urgency remission.

## Results

### Participants

The modified intent-to-treat population comprised 1,162 participants, of whom 868 received mirikizumab 300 mg intravenously every 4 weeks and 294 received placebo. Median age was 41 years (range 18 to 79). Most participants were White (71.7%) or Asian (25.0%), and 40.2% were female (Table 1).

**Table 1:** Demographics of the study participants in LUCENT-1

	N = 1162
Sex, n (%)	
Female	467 (40.2)
Male	695 (59.8)
Age (years)	
Mean (SD)	42.5 (13.9)
Median (range)	41 (18–79)
Age category, n (%)	
< 65 years	1071 (92.2)
≥ 65 years	91 (7.8)
Race, n (%)	
American Indian or Alaska Native	12 (1.0)
Asian	291 (25.0)
Black or African American	12 (1.0)
Native Hawaiian or Other Pacific Islander	1 (0.1)
White	833 (71.7)
Multiple	3 (0.3)
Missing	10 (0.9)

### Distribution of Urgency NRS scores

**Fig. 2:**
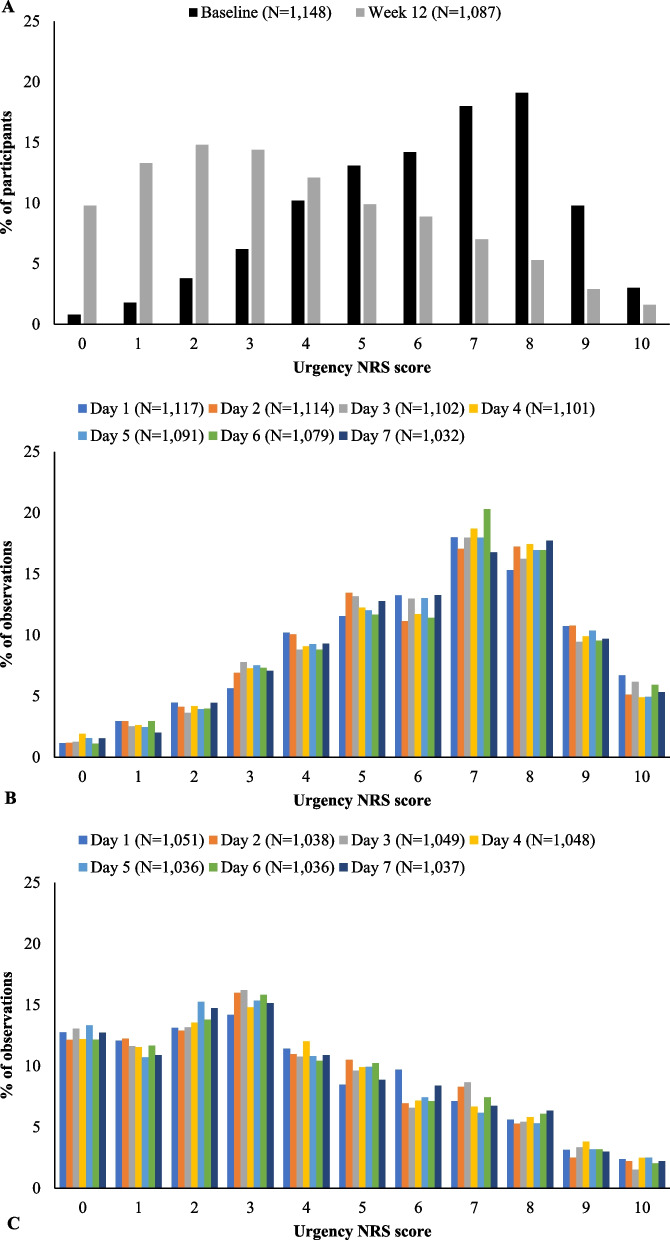
Urgency NRS score distributions. **A** Average weekly scores, rounded to the nearest whole number. **B** Daily scores recorded in the 7 days prior to the baseline clinical visit. **C** Daily scores recorded in the 7 days prior to the week 12 clinical visit

Collectively, participants registered the full range of weekly average Urgency NRS scores (0 to 10) at baseline and at week 12 (Fig. 2A). The mean (standard deviation) weekly Urgency NRS score was 6.2 (2.2) at baseline and 3.7 (2.6) at week 12. Median NRS score was also higher at baseline than at week 12 (6 vs. 3). The proportion of participants registering a score of 0 was 0.8% at baseline and 9.8% at week 12. A score of 10 was registered by 3.0% of participants at baseline and 1.6% at week 12. Figure 2B, C present the distributions of daily Urgency NRS scores in the 7 days prior to the baseline and week 12 visits, respectively. The distributions of daily scores were relatively uniform across days prior to both visits. There was therefore no evidence of any floor or ceiling effects among the weekly or daily values for the Urgency NRS at baseline or week 12. This suggests that weekly averages were appropriate to summarize daily Urgency NRS scores.

### Test–retest reliability

The ICC(2,1) was estimated to be 0.89 (95% CI 0.87, 0.90) among stable participants with no change in PGRS score between screening and baseline and 0.76 (0.70, 0.82) among stable participants who registered “no change” on the PGRC at week 4 (Table 2). This indicated that the Urgency NRS had strong test–retest reliability.

**Table 2:** Test–retest reliability of the urgency NRS

	Stable on PGRS^a^	Stable on PGRC^b^
Urgency NRS score		
n	642	202
Mean (SD)		
Screening	6.1 (2.10)	–
Baseline	6.2 (2.16)	6.3 (2.29)
Week 4	–	6.0 (2.28)
*p* value^c^	0.189	0.007
ICC(2,1) (95% CI)^d^	0.89 (0.87, 0.90)	0.76 (0.70, 0.82)

*CI* confidence interval, *ICC* intraclass correlation coefficient, *NRS* Numeric Rating Scale, *PGRC* Patient Global Rating of Change, *PGRS* Patient Global Rating of Severity, SD standard deviation

^a^Participants recording the same PGRS score at screening and baseline

^b^Participants recording no change on the PGRC at week 4

^c^Paired *t* test

^d^ICC(2,1) was calculated from a linear mixed-effects model with random intercepts using Urgency NRS scores from the two time points included in the analysis. A value ≥ 0.70 was considered evidence of acceptable test–retest reliability. Confidence intervals were constructed according to Cappelleri and Ting (2002)

### Known-groups validity

When the participant sample was dichotomized based on the median baseline PGRS score of 4, the mean Urgency NRS score at baseline was higher for participants with a PGRS score above the median (7.5) than for those with a PGRS score less than or equal to the median (5.4; LS mean difference 2.1; *p* < 0.0001) (Table 3). Cohen’s d for the difference between PGRS groups was 1.07, indicating a large standardized mean difference in baseline Urgency NRS scores between PGRS groups at baseline. Thus, mean Urgency NRS scores at baseline were consistently higher (worse) for participants with more severe self-rated overall UC symptoms than for those with less severe self-rated UC.

**Table 3:** Known-groups validity of the Urgency NRS at baseline and Week 12

	Urgency NRS score						
	n	Mean (SD)	Median (range)	LS mean (SE)^a^	LS mean difference (95% CI)^a^	*p* value^a^	Cohen’s d^b^
*Baseline*							
Median PGRS score at baseline							
≤ median (4)^c^	713	5.4 (2.05)	5 (0–10)	5.4 (0.07)	–	–	–
> median (4)^c^	435	7.5 (1.71)	8 (0–10)	7.5 (0.09)	2.1 (1.8, 2.3)	< 0.0001	1.07
*Week 12*							
PGRS score at week 12							
≤ 3	638	2.5 (1.98)	2 (0–10)	2.6 (0.08)	–	–	–
> 3	448	5.4 (2.32)	4 (0–10)	5.3 (0.09)	2.7 (2.4, 2.9)	< 0.0001	1.39
Clinical remission at week 12^d^							
Yes	246	2.2 (1.92)	2 (0–9)	2.3 (0.14)	–	–	–
No	833	4.1 (2.59)	4 (0–10)	4.1 (0.08)	1.8 (1.5, 2.2)	< 0.0001	0.79
Clinical response at week 12							
Yes	670	2.8 (2.24)	2 (0–10)	2.8 (0.08)	–	–	–
No	409	5.1 (2.46)	5 (0–10)	5.2 (0.10)	2.3 (2.1, 2.6)	< 0.0001	1.00

*CI* confidence interval, *LS* least-square, *NRS* Numeric Rating Scale, *PGRS* Patient Global Rating of Severity, SD standard deviation, *SE* standard error

^a^Derived from analysis of variance models that included Urgency NRS score as the dependent variable and PGRS subgroup as the independent variable

^b^Derived as the mean difference in Urgency NRS between known groups divided by the pooled standard deviation of the Urgency NRS scores at the given time point

^c^The median PGRS score at baseline was 4. The median PGRS score at week 12 was 3

^d^Clinical remission of UC was defined as a Mayo stool frequency subscore of 0, or 1 with a ≥ 1-point decrease from baseline; a Mayo rectal bleeding subscore of 0; and a Mayo endoscopic subscore of 0 or 1 (excluding friability)

^e^Decrease in MMS total score from baseline of ≥ 2 points and ≥ 30%; and Mayo rectal bleeding subscore 0 or 1 or decreased by ≥ 1 point from baseline

Similarly, Urgency NRS scores at week 12 were significantly higher among patients with PGRS greater than the median score of 3 (5.4 vs. 2.5; LS mean difference = 2.7; *p* < 0.0001), patients without a clinical response (5.1 vs. 2.8; LS mean difference = 1.8; *p* < 0.0001), and patients not in clinical remission at week 12 (4.1 vs. 2.2; LS mean difference = 2.3; *p* < 0.0001). Cohen’s d for the mean Urgency NRS at week 12 between known groups was 1.39 by PGRS, 0.79 by clinical remission, and 1.00 by clinical response status. Known-groups validity was also demonstrated based on uncollapsed PGRS score changes (Additional file 1: Table S1). These results indicate that the Urgency NRS demonstrated good known-groups validity at baseline and week 12.

### Convergent and discriminant validity

Correlations between Urgency NRS score and IBDQ total score and domain scores were moderate at baseline (− 0.31 to − 0.42) and moderate to large week 12 (− 0.46 to − 0.60) (Table 4). Large correlations were also observed with the PGRS at baseline (0.56) and week 12 (0.67) and with the PGRC at week 12 (0.52). Correlations with Mayo stool frequency were moderate at baseline (0.30) and moderate to large (0.49) at Week 12. Correlations with Mayo rectal bleeding were small to moderate at baseline (0.28) and moderate at week 12 (0.39). The Urgency NRS therefore demonstrated convergent validity. Conversely, correlations were very weak at baseline and weak to moderate at week 12 for the Urgency NRS with the objective Geboes score (0.02 at baseline and 0.28 at week 12) and Mayo endoscopic subscore (0.07 at baseline and 0.33 at week 12). The Urgency NRS therefore also demonstrated discriminant validity.

**Table 4:** Convergent and discriminant validity of the Urgency NRS at baseline and week 12

	Baseline			Week 12		
	n	Spearman correlation coefficient ^a^	95% CI	n	Spearman correlation coefficient ^a^	95% CI
IBDQ						
Bowel symptoms	1136	− 0.42	(− 0.47, − 0.37)	1079	− 0.60	(− 0.64, − 0.56)
Systemic symptoms	1136	− 0.37	(− 0.42, − 0.32)	1079	− 0.50	(− 0.55, − 0.46)
Emotional functioning	1136	− 0.31	(− 0.37, − 0.26)	1079	− 0.46	(− 0.51, − 0.42)
Social functioning	1136	− 0.38	(− 0.42, − 0.32)	1079	− 0.50	(− 0.54, − 0.45)
Total score	1136	− 0.41	(− 0.45, − 0.36)	1079	− 0.57	(− 0.61, − 0.53)
PGRS	1148	0.56	(0.52, 0.60)	1086	0.67	(0.63, 0.70)
PGRC^b^	–	–	–	1079	0.52	(0.47, 0.56)
MMS						
Stool frequency	1148	0.30	(0.25, 0.35)	1087	0.49	(0.44, 0.53)
Rectal bleeding	1 148	0.28	(0.22, 0.33)	1087	0.39	(0.33, 0.44)
Endoscopic subscore	1147	0.07	(0.01, 0.13)	1079	0.33	(0.27, 0.38)
Total score	1147	0.36	(0.31, 0.41)	1079	0.52	(0.47, 0.56)
Geboes score	1,128	0.02	(− 0.04, 0.08)	1064	0.28	(0.23, 0.34)

*CI* confidence interval, *IBDQ* Inflammatory Bowel Disease Questionnaire, *MMS* modified Mayo score, *NRS* Numeric Rating Scale, *PGRC* Patient Global Rating of Change, *PGRS* Patient Global Rating of Severity

^a^ > 0.5 = strong, 0.3 to 0.5 = moderate, and < 0.3 = weak

^b^Not assessed at baseline

### Responsiveness

Decreases (improvements) in Urgency NRS scores at week 12 were higher in participants who achieved clinical remission than in those with active disease (LS mean change from baseline − 3.8 vs. − 2.0; effect size (ES) = 0.80; *p* < 0.0001) (Table 5). Similarly, decreases in Urgency NRS scores were higher in clinical responders than in non-responders (LS mean change from baseline − 3.3 vs. − 1.0; ES = 1.07; *p* < 0.0001). Decreases in Urgency NRS scores were also higher in participants achieving IBDQ remission (LS mean change from baseline − 3.3 vs. − 1.3; ES = 0.70; *p* < 0.0001) and in participants with a week 12 PGRS score less than or equal to the median (LS mean change from baseline − 3.5 vs. − 0.9; ES = 1.05; *p* < 0.0001). Responsiveness was also demonstrated based on uncollapsed PGRS score changes (Additional file 1: Table S2) and uncollapsed PGRC categories (Additional file 1: Table S3). Collectively, these findings indicate that the Urgency NRS was able to detect changes in bowel urgency in patients whose UC severity and quality of life changed at week 12.

**Table 5:** Responsiveness at week 12 based on clinical remission, clinical response, IBDQ remission, and median PGRS score

	Clinical remission^a^		Clinical response^b^		IBDQ remission^c^		PGRS score^d^	
	No	Yes	No	Yes	No	Yes	> median	≤ median
n	833	246	409	670	474	605	448	638
Mean	− 2.0	− 3.8	− 1.0	− 3.3	− 1.6	− 3.1	− 1.1	− 3.4
Standard deviation	2.47	2.37	2.16	2.36	2.37	2.49	2.10	2.42
Range	− 10 to + 7	− 10 to + 2	− 8 to + 7	− 10 to + 3	− 9 to + 7	− 10 to + 6	− 8 to + 7	− 10 to + 3
Effect size^f^	-	0.80	-	1.07	-	0.70	-	1.06
LS mean change from baseline (SE)^e^	− 2.0 (0.08)	− 3.8 (0.14)	− 1.0 (0.10)	− 3.3 (0.08)	− 1.3 (0.10)	− 3.3 (0.09)	− 0.9 (0.09)	− 3.5 (0.08)
LS mean difference (SE)^e^	–	1.8 (0.16)	–	2.3 (0.13)	–	2.0 (0.13)	–	2.7 (0.12)
*p *value^e^	–	< 0.0001	–	< 0.0001	–	< 0.0001	–	< 0.0001

*IBDQ* Inflammatory Bowel Disease Questionnaire, *LS* least-square, *NRS* Numeric Rating Scale, *PGRS* Patient Global Rating of Severity, *SE* standard error

^a^Clinical remission of UC was defined as a Mayo stool frequency subscore of 0, or 1 with a ≥ 1-point decrease from baseline; a Mayo rectal bleeding subscore of 0; and a Mayo endoscopic subscore of 0 or 1 (excluding friability)

^b^Decrease in MMS total score from baseline of ≥ 2 points and ≥ 30%; and Mayo rectal bleeding subscore 0 or 1 or decreased by ≥ 1 point from baseline

^c^IBDQ remission was defined as an IBDQ total score ≥ 170

^d^The median PGRS score at week 12 was 3

^e^Derived from one-way analysis of covariance models with change in urgency NRS score as the dependent variable, and baseline Urgency NRS score and meaningful improvement subgroup for the anchor (Yes/No) as independent variables

^f^Calculated as the mean difference in change from baseline to week 12 on the Urgency NRS between response groups divided by the pooled standard deviation of the Urgency NRS scores at baseline

### Meaningful within-patient improvement from baseline

**Fig. 3:**
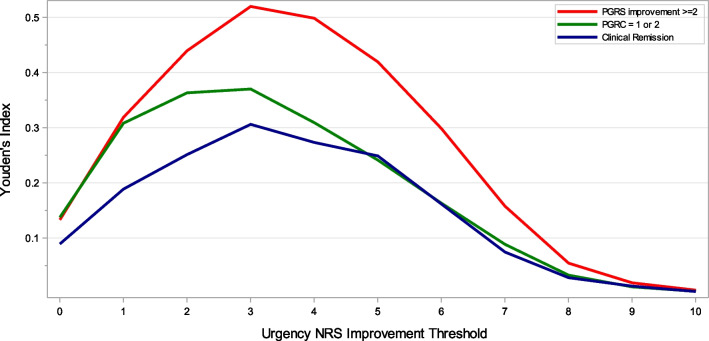
Youden’s Index from an anchor-based analysis of improvement in Urgency NRS from baseline to week 12. Clinical remission was defined as a Mayo stool frequency subscore of 0, or 1 with a ≥ 1-point decrease from baseline; a Mayo rectal bleeding subscore of 0; and a Mayo endoscopic subscore of 0 or 1 (excluding friability)

The threshold with the maximum YI and AUROC for predicting a ≥ 2 point PGRS improvement was a 3-point Urgency NRS improvement (YI = 0.52, AUROC = 0.76) (Table 6, Fig. 3). A ≥ 3-point improvement on the Urgency NRS therefore yields the best balance between sensitivity and specificity of any Urgency NRS threshold at identifying large improvement in overall symptom severity based on the PGRS. A 3-point threshold for Urgency NRS improvement also maximized Youden’s index and AUROC for the PGRC (YI = 0.37, AUROC = 0.69). When clinical remission was used as the anchor, YI and AUROC were maximized (YI = 0.31, AUROC = 0.65) at an Urgency NRS improvement threshold of 3 points (Fig. 3 and Additional file 1: Table S4), indicating that a ≥ 3-point improvement on the Urgency NRS best corresponds to patients achieving clinical remission. Collectively, these analyses suggest that a ≥ 3-point improvement on the Urgency NRS represents a meaningful within-patient improvement in bowel urgency in moderate-to-severe UC patients.

**Table 6:** Anchor-based analysis of meaningful change from baseline to week 12: PGRS and PGRC

Urgency NRS score change threshold^a^	PGRS^b^						PGRC^c^					
	Sensitivity	Specificity	Positive predictive value	Negative predictive value	Youden’s index^d^	AUROC^e^	Sensitivity	Specificity	Positive predictive value	Negative predictive value	Youden’s index^d^	AURC ^e^
+ 10	1.00	0.00	0.35	**-**	0.00	**-**	1.00	0.00	0.58	**-**	0.00	-
+ 9	1.00	0.00	0.35	**-**	0.00	**-**	1.00	0.00	0.58	**-**	0.00	-
+ 8	1.00	0.00	0.35	**-**	0.00	**-**	1.00	0.00	0.58	**-**	0.00	-
+ 7	1.00	0.00	0.35	**-**	0.00	**-**	1.00	0.00	0.58	**-**	0.00	-
+ 6	1.00	0.00	0.35	1.00	0.00	0.50	1.00	0.00	0.58	1.00	0.00	0.50
+ 5	1.00	0.01	0.35	1.00	0.01	0.50	1.00	0.01	0.58	0.75	0.01	0.50 ara>
+ 4	1.00	0.01	0.35	1.00	0.01	0.50	1.00	0.01	0.58	0.67	0.01	0.50
+ 3	1.00	0.01	0.35	1.00	0.01	0.51	1.00	0.01	0.58	0.71	0.01	0.50
+ 2	1.00	0.03	0.35	1.00	0.03	0.51	1.00	0.03	0.58	0.83	0.03	0.51
+ 1	1.00	0.06	0.36	0.95	0.05	0.53	0.99	0.07	0.59	0.81	0.06	0.53
0	0.99	0.14	0.38	0.96	0.13	0.57	0.96	0.18	0.62	0.76	0.14	0.57
− 1	0.97	0.35	0.44	0.95	0.32	0.66	0.89	0.42	0.68	0.74	0.31	0.65
− 2	0.90	0.54	0.51	0.91	0.44	0.72	0.77	0.59	0.72	0.65	0.36	0.68
− 3	0.79	0.74	0.61	0.87	**0.52**	**0.76**	0.60	0.77	0.78	0.59	**0.37**	**0.69**
− 4	0.65	0.85	0.70	0.82	0.50	0.75	0.45	0.86	0.81	0.53	0.31	0.66
− 5	0.48	0.94	0.80	0.77	0.42	0.71	0.31	0.93	0.86	0.50	0.24	0.62
− 6	0.39	0.96	0.82	0.73	0.30	0.65	0.21	0.95	0.85	0.47	0.16	0.58
− 7	0.17	0.99	0.88	0.69	0.16	0.58	0.11	0.98	0.89	0.45	0.09	0.54
− 8	0.06	1.00	0.88	0.67	0.05	0.53	0.04	1.00	0.92	0.43	0.03	0.52
− 9	0.02	1.00	1.00	0.66	0.02	0.51	0.01	1.00	1.00	0.43	0.01	0.51
− 10	0.01	1.00	1.00	0.66	0.01	0.50	0.00	1.00	1.00	0.42	0.00	0.50

*AUROC* area under the receiver operating characteristic curve, *NRS* Numeric Rating Scale, *PGRC* Patient Global Rating of Change, *PGRS* Patient Global Rating of Severity

^a^An increase in Urgency NRS score represents deterioration and a decrease represents improvement

^b^PGRS responders were participants with a ≥ 2-point improvement in PGRS score between baseline and week 12

^c^PGRC responders were participants with a PGRC score of 1 or 2 at week 12

^d^Sensitivity + specificity − 1. Bold: highest value of Youden’s index. A higher value of Youden’s index indicates a better balance of sensitivity and specificity of the potential NRS score change threshold in identifying responders based on the anchor (PGRS or PGRC)

^e^AUROC was calculated from a logistic regression model with the anchor variable as the dependent variable and urgency improvement status (as defined by the Urgency NRS threshold) as the independent variable. Bold: highest value of AUROC

### Threshold for bowel urgency remission

**Fig. 4:**
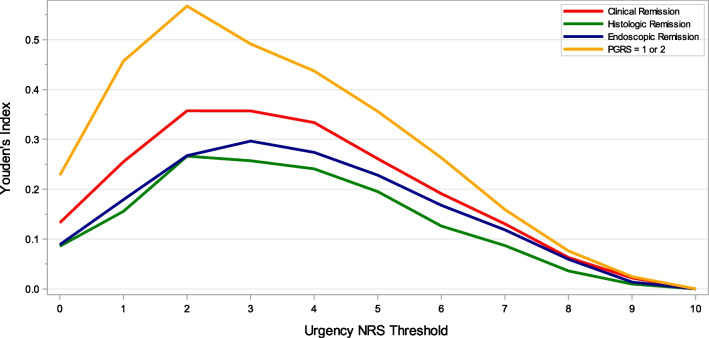
Youden’s Index from an anchor-based analysis of remission on the Urgency NRS at week 12. Clinical remission was defined as a Mayo stool frequency subscore of 0, or 1 with a ≥ 1-point decrease from baseline; a Mayo rectal bleeding subscore of 0; and a Mayo endoscopic subscore of 0 or 1 (excluding friability). Histologic remission was defined as a Geboes histologic score of 2b. Endoscopic remission was defined as a Mayo endoscopic subscore of 0 or 1 (excluding friability)

A threshold for bowel urgency remission—associated with clinical remission or inactive disease—was also explored by conducting anchor-based analyses with remission endpoints as anchor variables. An Urgency NRS threshold of 2 points or lower yielded the highest YI and AUROC with patients achieving UC symptom remission based on the PGRS (YI = 0.57, AUROC = 0.78), clinical remission (YI = 0.34, AUROC = 0.67), and histologic remission (YI = 0.25, AUROC = 0.62) (Table 7, Fig. 4, and Additional file 1: Table S5). For these three anchors, an Urgency NRS threshold of 1 point had lower YI and sensitivity than a threshold of 2 points, but higher specificity and higher or comparable positive predictive value. For endoscopic remission based on the Mayo endoscopic subscore, YI and AUROC were maximized at an Urgency NRS threshold of 3 points (YI = 0.27, AUROC = 0.63) and was also high with a threshold of 2 points (YI = 0.25, AUROC = 0.62) (Fig. 4 and Additional file 1: Table S5). Compared to a threshold of 2 points, a threshold of 1 point had lower values for YI and sensitivity but higher specificity and marginally higher positive predictive value. Collectively, these findings suggest that an Urgency NRS score of ≤ 2 points was best associated with patients achieving symptom, clinical, endoscopic, or histologic remission. An Urgency NRS score of ≤ 1 point would be a more conservative definition to identify patients with bowel urgency remission. Compared to a definition of ≤ 2 points, a definition of ≤ 1 point had lower YI but was superior in terms of specificity and generally superior in terms of positive predictive value.

**Table 7:** Anchor-based analysis of urgency remission at week 12: PGRS and clinical remission of UC

Urgency NRS score threshold^a^	PGRS^b^						Clinical remission^c^					
	Sensitivity	Specificity	Positive predictive value	Negative predictive value	Youden’s index^d^	AUROC^e^	Sensitivity	Specificity	Positive predictive value	Negative predictive value	Youden’s index^d^	AUROC^e^
0	0.26	0.98	0.84	0.74	0.23	0.62	0.20	0.93	0.46	0.80	0.13	0.57
1	0.54	0.92	0.75	0.81	0.46	0.73	0.42	0.82	0.41	0.83	0.25	0.62
2	0.76	0.80	0.64	0.88	**0.57**	**0.78**	0.64	0.70	0.38	0.87	**0.34**	**0.67**
3	0.85	0.63	0.52	0.90	0.48	0.74	0.78	0.55	0.34	0.90	0.33	0.67
4	0.93	0.49	0.46	0.94	0.42	0.71	0.88	0.43	0.31	0.92	0.31	0.65
5	0.97	0.36	0.42	0.96	0.33	0.67	0.92	0.31	0.28	0.93	0.23	0.62
6	0.99	0.24	0.38	0.99	0.24	0.62	0.96	0.20	0.26	0.94	0.16	0.58
7	1.00	0.14	0.35	0.99	0.14	0.57	0.99	0.12	0.25	0.97	0.11	0.56
8	1.00	0.07	0.33	1.00	0.07	0.53	1.00	0.06	0.24	0.98	0.05	0.53
9	1.00	0.02	0.33	1.00	0.02	0.51	1.00	0.02	0.23	1.00	0.02	0.51
10	1.00	0.00	0.32	**-**	0.00	**-**	1.00	0.00	0.23	**-**	0.00	-

*AUROC* area under the receiver operating characteristic curve, *NRS* Numeric Rating Scale, *PGRS* Patient Global Rating of Severity, *UC* ulcerative colitis

^a^A higher Urgency NRS score represents more severe urgency

^b^UC symptom remission was defined as a PGRS score of 1 or 2

^c^Clinical remission of UC was defined as a Mayo stool frequency subscore of 0, or 1 with a ≥ 1-point decrease from baseline; a Mayo rectal bleeding subscore of 0; and a Mayo endoscopic subscore of 0 or 1 (excluding friability)

^d^Sensitivity + specificity − 1. Bold: highest value of Youden’s index. A higher value of Youden’s index indicates a better balance of sensitivity and specificity of the potential NRS score threshold in identifying participants with UC remission based on the anchor (PGRS or clinical remission)

^e^AUROC was calculated from a logistic regression model with the anchor variable as the dependent variable and urgency remission status (as defined by the Urgency NRS threshold) as the independent variable. Bold: highest value of AUROC

## Discussion

The present analysis used data from a phase 3 clinical trial to assess the measurement properties of the Urgency NRS, a content-valid PRO measure for capturing changes in the severity of bowel urgency in patients with UC [5]. The Urgency NRS showed good test–retest reliability based on data for stable participants without change on the PGRS and PGRC, and good known-groups validity based on PGRS score categories. It also showed convergent and discriminant validity. Correlations between the Urgency NRS and other assessments were stronger at week 12 than at baseline, presumably due to variability in patient outcomes resulting from different levels of response to active treatment versus placebo. Furthermore, the Urgency NRS was responsive to changes in UC severity.

For an NRS-based PRO measure to be useful in clinical trials, one must be able to interpret scores and score changes on the scale. This study found that an Urgency NRS score improvement of ≥ 3 points is clinically meaningful for patients with moderately to severely active UC and that an Urgency NRS score of ≤ 1 point represents bowel urgency remission. For three of the four anchors included in the bowel urgency remission analysis, both Youden’s index and area under the receiver operating characteristic curve were maximized at an Urgency NRS threshold of 2 points. Compared to a remission threshold of ≤ 2 points, an Urgency NRS threshold of ≤ 1 point represents a more conservative definition for bowel urgency remission and benefits from higher positive predictive value and higher specificity.

These results support the notion that patients in UC remission or with inactive disease may still register an Urgency NRS score greater than 0. In qualitative interviews with 19 patients with moderately to severely active UC, participants indicated that an Urgency NRS score of 1 to 3 reflected mild bowel urgency with minimal impact on daily life [30]. In addition, while a score of 0 on the Urgency NRS is defined as “no urgency,” a certain level of variability in bowel urgency should be expected, especially considering that bowel urgency can occur in healthy people without underlying inflammation [31] and that patients completed the Urgency NRS daily over a prolonged period of time. Therefore, achieving a mean score of 0 on this 11-point NRS scale may be an unrealistic treatment target. In the present analysis, we showed that an Urgency NRS score of up to 2 was most associated with achieving clinical remission, endoscopic remission, histologic remission, and resolution or very minimal overall symptom severity according to the PGRS. These results support the notion that patients in remission or inactive disease may still report minimal residual levels of bowel urgency on the Urgency NRS that they consider “normal.” This is in line with observations from the recent Study of a Prospective Adult Research Cohort with Inflammatory Bowel Disease (SPARC-IBD), where 39% of UC patients with mild urgency and 9% with moderate-to-severe urgency reported no abdominal pain, no bleeding, and normal bowel frequency [32].

Other PRO instruments for capturing bowel urgency include the Patient Simple Clinical Colitis Activity Index (P-SCCAI) [33] and Ulcerative Colitis Patient-Reported Outcomes (UC-PRO) [34], validated multi-item instruments with individual items on bowel urgency. Also available are the Symptoms and Impacts Questionnaire for Ulcerative Colitis (SIQ-UC) [35] and Crohn’s and Ulcerative Colitis Questionnaire (CUCQ) [36, 37], which were developed to capture the symptoms and impacts of UC, including bowel urgency; and an unvalidated single-item measure used in SPARC-IBD [32]. However, the UC-PRO and CUCQ capture the frequency but not severity of bowel urgency. The CUCQ is further limited by its 2-week recall period, meaning that it is unable to capture daily fluctuations in symptoms. The P-SCCAI and SIQ-UC capture the severity of bowel urgency, but respectively use a binary response option and a 5-point response scale. By comparison, the Urgency NRS’s 11-point scale allows changes in bowel urgency to be better captured through a wider range of scores.

The validation work was conducted in accordance with current standards for evaluating the psychometric properties of PRO instruments [17, 29, 38–40] using a large patient sample. Another strength is that the analyses of thresholds for meaningful change and urgency remission included both PROs and objective clinical outcomes based on blinded assessments. Also, generalizability of the findings is enhanced by the inclusion of patients from a wide range of geographies, with similar demographics as those in other recent UC trials [41, 42]. However, there were very few Black participants in the trial so generalizability to this population remains to be tested.

One limitation of this validation study is that the psychometric evaluation only used weekly average Urgency NRS scores collected daily with 24-h recall periods. This reflected the intended use of the Urgency NRS in clinical trials. Psychometric properties for a one-time administration of the Urgency NRS with a longer recall period, which may be more applicable to clinical practice or real-world studies, was not examined. However, the psychometric properties of the Urgency NRS should, in theory, be very similar for a single assessment with a 7-day recall period as for a weekly average of daily scores. Minimal differences are seen in the distributions of Urgency NRS scores between daily and one-time assessments (as illustrated in Fig. 2), and the mean change from baseline should also be similar. As a result, Spearman correlations for convergent and discriminant validity and effect sizes for known-groups validity and responsiveness should be very similar. Assessments with longer recall periods have been shown to give higher estimates of ICC [43, 44]. Because high ICC values were seen among daily administration of the Urgency NRS, we should also expect high ICC values for one-time administration. Given the consistent results from the anchor-based analyses of meaningful within-patient improvement and bowel urgency remission, we believe the definitions for these endpoints would similarly hold for one-time administration of the Urgency NRS with a 1-week recall period, although this will require testing in future studies.

A limitation of using diagnostic test statistics (sensitivity, specificity, Youden’s Index, and AUROC) to define meaningful within-patient improvement and minimal to no bowel urgency is that they may only be applicable to the current sample; their generalizability to other samples or populations is unconfirmed. It would therefore be beneficial for future studies to reproduce or confirm our findings in other UC patient cohorts.

## Conclusions

Improvement in the severity of bowel urgency is an important outcome to capture in UC clinical trials. We have developed and validated the Urgency NRS as a new PRO instrument for capturing changes in bowel urgency severity in patients with UC. The good psychometric properties of the Urgency NRS indicate that it can be used in clinical trials to evaluate treatment benefits in patients with moderately to severely active UC, and potentially in routine clinical practice.

## Supplementary Information

**Additional file 1. Supplementary Table 1**. Known-groups validity based on uncollapsed PGRS scores.** Supplementary Table 2**. Responsiveness at week 12 based on uncollapsed PGRS score changes.** Supplementary Table 3**. Responsiveness at week 12 based on uncollapsed PGRC categories.** Supplementary Table 4**. Anchor-based analysis of meaningful change from baseline: clinical remission.** Supplementary Table 5**. Anchor-based analysis of urgency remission at week 12: endoscopic remission and histologic remission.

## Data Availability

The datasets used and/or analysed during the current study are available from the corresponding author on reasonable request.

## Abbreviations

CI: Confidence interval
CUCQ: Crohn’s and Ulcerative Colitis Questionnaire
eDiary: Electronic diary
IBDQ: Inflammatory Bowel Disease Questionnaire
ICC: Intraclass correlation coefficient
LS: Least-square
MMS: Modified Mayo score
NRS: Numeric rating scale
PGRC: Patient Global Rating of Change
PGRS: Patient Global Rating of Severity
PRO: Patient-reported outcome
P-SCCAI: Patient Simple Clinical Colitis Activity Index
SD: Standard deviation
SE: Standard error
SIQ-UC: Symptoms and Impacts Questionnaire for Ulcerative Colitis
SPARC IBD: Study of a Prospective Adult Research Cohort with Inflammatory Bowel Disease
UC: Ulcerative colitis
UC-PRO: Ulcerative Colitis Patient-Reported Outcomes
