# Supplementary material for: Psychometric evaluation of the Urgency NRS as a new patient-reported outcome measure for patients with ulcerative colitis
Source: J Patient Rep Outcomes. 2022 Nov 5;6:114. doi: 10.1186/s41687-022-00522-2 (PMC9637076; doi:10.1186/s41687-022-00522-2)
Supplement: Supplementary file 1 — Additional file 1. Supplementary Table 1. Known-groups validity based on uncollapsed PGRS scores. Supplementary Table 2. Responsiveness at week 12 based on uncollapsed PGRS score changes. Supplementary Table 3. Responsiveness at week 12 based on uncollapsed PGRC categories. Supplementary Table 4. Anchor-based analysis of meaningful change from baseline: clinical remission. Supplementary Table 5. Anchor-based analysis of urgency remission at week 12: endoscopic remission and histologic remission. [file 41687_2022_522_MOESM1_ESM.docx]

# Supplementary Material

**Supplementary Table 1. Known-groups validity based on uncollapsed PGRS scores**

|  |  | | **Baseline urgency NRS score** | | | | | |
| --- | --- | --- | --- | --- | --- | --- | --- | --- |
|  | **n** | **Mean (SD)** | | **Median (range)** | **LS mean (SE) ^a^** | **LS mean difference (95% CI) ^a b^** | **Cohen’s D** | **p-value ^a b^** |
| PGRS score at baseline |  |  | |  |  |  |  |  |
| 1 (None) or 2 (Very mild) | 30 | 2.4 (1.43) | | 2 (0–6) | 2.4 (0.33) | - |  | - |
| 3 (Mild) | 127 | 4.2 (2.05) | | 4 (0–9) | 4.2 (0.16) | - |  | - |
| 4 (Moderate) | 556 | 5.8 (1.82) | | 6 (1–10) | 5.8 (0.08) | - |  | - |
| 5 (Severe) | 390 | 7.3 (1.65) | | 8 (0–10) | 7.3 (0.09) | - |  | - |
| 6 (Very severe) | 45 | 8.5 (1.88) | | 9 (1–10) | 8.5 (0.27) | - |  | - |
| Comparison of PGRS subgroups |  |  | |  |  |  |  |  |
| 6 vs. 5 | - | - | | - | - | 1.1 (0.3, 2.0) | 0.56 | 0.0029 |
| 6 vs. 4 | - | - | | - | - | 2.6 (1.8, 3.5) | 1.33 | <0.0001 |
| 6 vs. 3 | - | - | | - | - | 4.3 (3.4, 5.3) | 2.19 | <0.0001 |
| 6 vs. 1 or 2 | - | - | | - | - | 6.1 (4.8, 7.4) | 3.11 | <0.0001 |
| 5 vs. 4 | - | - | | - | - | 1.5 (1.2, 1.9) | 0.77 | <0.0001 |
| 5 vs. 3 | - | - | | - | - | 3.2 (2.6, 3.7) | 1.63 | <0.0001 |
| 5 vs. 1 or 2 | - | - | | - | - | 5.0 (3.9, 6.0) | 2.55 | <0.0001 |
| 4 vs. 3 | - | - | | - | - | 1.7 (1.1, 2.2) | 0.87 | <0.0001 |
| 4 vs. 1 or 2 | - | - | | - | - | 3.5 (2.4, 4.5) | 1.79 | <0.0001 |
| 3 vs. 1 or 2 | - | - | | - | - | 1.8 (0.7, 2.9) | 0.92 | <0.0001 |

^a^ Derived from an analysis of variance model that included Urgency NRS score as the dependent variable and PGRS subgroup as the independent variable.

^b^ Corrected for multiple comparisons by Scheffe’s method.

CI, confidence interval; LS, least-square; NRS, Numeric Rating Scale; PGRS, Patient Global Rating of Severity; SD, standard deviation; SE, standard error.

**Supplementary Table 2. Responsiveness at week 12 based on uncollapsed PGRS score changes**

|  | **PGRS score change ^a^** | | | | | |
| --- | --- | --- | --- | --- | --- | --- |
|  | **4-point decrease** | **3-point decrease** | **2-point decrease** | **1-point decrease** | **No change** | **1-point increase** |
| n | 39 | 113 | 224 | 345 | 313 | 52 |
| Mean | −6.7 | −5.2 | −3.6 | −2.3 | −0.7 | +0.5 |
| Standard deviation | 1.58 | 1.77 | 2.01 | 1.96 | 1.67 | 2.40 |
| Range | −10 to −2 | -9 to −1 | −10 to +2 | −8 to +7 | −6 to +6 | −6 to +7 |
| LS mean change from baseline (SE) ^b^ | −6.2 (0.28) | −5.0 (0.16) | −3.5 (0.12) | −2.3 (0.09) | −0.8 (0.10) | +0.3 (0.24) |
| LS mean difference (95% CI) ^b c^ |  |  |  |  |  |  |
| vs. 4-point decrease | - | 1.2 (0.1, 2.3) | 2.7 (1.7, 3.7) | 3.8 (2.9, 4.8) | 5.3 (4.4, 6.3) | 6.5 (5.3, 7.7) |
| vs. 3-point decrease | - | - | 1.5 (0.8, 2.2) | 2.6 (2.0, 3.3) | 4.1 (3.5, 4.8) | 5.3 (4.4, 6.3) |
| vs. 2-point decrease | - | - | - | 1.1 (0.6, 1.6) | 2.6 (2.1, 3.1) | 3.8 (2.9, 4.7) |
| vs. 1-point decrease | - | - | - | - | 1.5 (1.1, 2.0) | 2.7 (1.8, 3.5) |
| vs. no change | - | - | - | - | - | 1.2 (0.3, 2.0) |
| p-value ^b c^ |  |  |  |  |  |  |
| vs. 4-point decrease | - | 0.0153 | <0.0001 | <0.0001 | <0.0001 | <0.0001 |
| vs. 3-point decrease | - | - | <0.0001 | <0.0001 | <0.0001 | <0.0001 |
| vs. 2-point decrease | - | - | - | <0.0001 | <0.0001 | <0.0001 |
| vs. 1-point decrease | - | - | - | - | <0.0001 | <0.0001 |
| vs. no change | - | - | - | - | - | 0.0010 |

^a^ An increase in PGRS score represents deterioration and a decrease represents improvement.

^b^ Derived from one-way analysis of covariance models with change in urgency NRS score as the dependent variable, and baseline Urgency NRS score and PGRS subgroup as independent variables.

^c^ Corrected for multiple comparisons by Scheffe’s method.

CI, confidence interval; LS, least-square; PGRS, Patient Global Rating of Severity; SE, standard error.

**Supplementary Table 3. Responsiveness at week 12 based on uncollapsed PGRC categories**

|  | **PGRC category** | | | | | | |
| --- | --- | --- | --- | --- | --- | --- | --- |
|  | **Very much better** | **Much better** | **A little better** | **No change** | **A little worse** | **Much worse** | **Very much worse** |
| n | 246 | 377 | 269 | 125 | 34 | 20 | 8 |
| Mean | −4.1 | −2.8 | −1.6 | −0.7 | −0.2 | −0.5 | +0.6 |
| Standard deviation | 2.36 | 2.30 | 2.11 | 1.96 | 1.81 | 2.68 | 4.60 |
| Range | −10 to +1 | −10 to +6 | −7 to +7 | −8 to +3 | −4 to +5 | −7 to +3 | −8 to +7 |
| LS mean change from baseline (SE) ^a^ | −4.1 (0.12) | −2.9 (0.10) | −1.5 (0.12) | −0.8 (0.17) | −0.2 (0.33) | +0.1 (0.44) | +0.8 (0.69) |
| LS mean difference (95% CI) ^a b^ |  |  |  |  |  |  |  |
| vs. Very much better | - | 1.2 (0.7, 1.8) | 2.6 (2.0, 3.2) | 3.4 (2.6, 4.2) | 4.0 (2.7, 5.2) | 4.2 (2.6, 5.9) | 4.9 (2.4, 7.4) |
| vs. Much better | - | - | 1.4 (0.8, 2.0) | 2.2 (1.4, 2.9) | 2.7 (1.5, 4.0) | 3.0 (1.4, 4.6) | 3.7 (1.2, 6.1) |
| vs. A little better | - | - | - | 0.8 (0.0, 1.5) | 1.3 (0.1, 2.6) | 1.6 (0.0, 3.2) | 2.3 (−0.2, 4.8) |
| vs. No change | - | - | - | - | 0.6 (−0.8, 1.9) | 0.9 (−0.8, 2.5) | 1.5 (−1.0, 4.0) |
| vs. A little worse | - | - | - | - | - | 0.3 (−1.7, 2.2) | 0.9 (−1.8, 3.7) |
| vs. Much worse | - | - | - | - | - | - | 0.6 (−2.3, 3.6) |
| p-value ^a b^ |  |  |  |  |  |  |  |
| vs. Very much better | - | <0.0001 | <0.0001 | <0.0001 | <0.0001 | <0.0001 | <0.0001 |
| vs. Much better | - | - | <0.0001 | <0.0001 | <0.0001 | <0.0001 | 0.0001 |
| vs. A little better | - | - | - | 0.0481 | 0.0309 | 0.0499 | 0.1092 |
| vs. No change | - | - | - | - | 0.8903 | 0.7701 | 0.6132 |
| vs. A little worse | - | - | - | - | - | 0.9996 | 0.9607 |
| vs. Much worse | - | - | - | - | - | - | 0.9958 |

^a^ Derived from one-way analysis of covariance models with change in urgency NRS score as the dependent variable, and baseline Urgency NRS score and PGRC subgroup as independent variables.

^b^ Corrected for multiple comparisons by Scheffe’s method.

CI, confidence interval; LS, least-square; PGRC, Patient Global Rating of Change; SE, standard error.

**Supplementary Table 4. Anchor-based analysis of meaningful change from baseline: clinical remission**

| **Urgency NRS score change threshold ^a^** | **Sensitivity** | **Specificity** | **Positive predictive value** | **Negative predictive value** | **Youden’s index ^b^** | **AUROC ^c^** |
| --- | --- | --- | --- | --- | --- | --- |
| +10 | 1.00 | 0.00 | 0.23 | **-** | 0.00 | - |
| +9 | 1.00 | 0.00 | 0.23 | **-** | 0.00 | - |
| +8 | 1.00 | 0.00 | 0.23 | **-** | 0.00 | - |
| +7 | 1.00 | 0.00 | 0.23 | **-** | 0.00 | - |
| +6 | 1.00 | 0.00 | 0.23 | 1.00 | 0.00 | 0.50 |
| +5 | 1.00 | 0.01 | 0.23 | 1.00 | 0.01 | 0.50 |
| +4 | 1.00 | 0.01 | 0.23 | 1.00 | 0.01 | 0.50 |
| +3 | 1.00 | 0.01 | 0.23 | 1.00 | 0.01 | 0.50 |
| +2 | 1.00 | 0.02 | 0.23 | 1.00 | 0.02 | 0.51 |
| +1 | 1.00 | 0.05 | 0.24 | 0.98 | 0.04 | 0.52 |
| 0 | 0.97 | 0.12 | 0.25 | 0.93 | 0.09 | 0.55 |
| −1 | 0.91 | 0.28 | 0.27 | 0.91 | 0.19 | 0.59 |
| −2 | 0.81 | 0.44 | 0.30 | 0.89 | 0.25 | 0.63 |
| −3 | 0.68 | 0.62 | 0.35 | 0.87 | **0.31** | **0.65** |
| −4 | 0.53 | 0.74 | 0.38 | 0.84 | 0.27 | 0.64 |
| −5 | 0.40 | 0.85 | 0.44 | 0.83 | 0.25 | 0.62 |
| −6 | 0.27 | 0.89 | 0.43 | 0.81 | 0.16 | 0.58 |
| −7 | 0.13 | 0.95 | 0.42 | 0.79 | 0.07 | 0.54 |
| −8 | 0.05 | 0.98 | 0.44 | 0.78 | 0.03 | 0.51 |
| −9 | 0.02 | 1.00 | 0.57 | 0.77 | 0.01 | 0.51 |
| −10 | 0.00 | 1.00 | 0.50 | 0.77 | 0.00 | 0.50 |

Clinical remission of UC was defined as a Mayo stool frequency subscore of 0 or 1 with a ≥1‑point decrease from baseline; a Mayo rectal bleeding subscore of 0; and a Mayo endoscopic subscore of 0 or 1 (excluding friability).

^a^ An increase in Urgency NRS score represents deterioration and a decrease represents improvement.

^b^ Sensitivity + specificity − 1. Bold: highest value of Youden’s index. A higher value of Youden’s index indicates a better balance of sensitivity and specificity of the potential NRS score change threshold in identifying responders based on clinical remission of UC.

^c^ AUROC was calculated from a logistic regression model with clinical remission as the dependent variable and urgency improvement status (as defined by the Urgency NRS threshold) as the independent variable. Bold: highest value of AUROC.

AUROC, area under the receiver operating characteristic curve; NRS, Numeric Rating Scale; UC, ulcerative colitis.

**Supplementary Table 5. Anchor-based analysis of urgency remission at week 12: endoscopic remission and histologic remission**

| **Urgency NRS score threshold ^a^** | **Endoscopic remission ^b^** | | | | | |  | **Histologic remission ^c^** | | | | | |
| --- | --- | --- | --- | --- | --- | --- | --- | --- | --- | --- | --- | --- | --- |
|  | **Sensitivity** | **Specificity** | **Positive predictive value** | **Negative predictive value** | **Youden’s index ^d^** | **AUROC ^e^** |  | **Sensitivity** | **Specificity** | **Positive predictive value** | **Negative predictive value** | **Youden’s index ^d^** | **AUROC ^e^** |
| 0 | 0.15 | 0.93 | 0.53 | 0.68 | 0.08 | 0.54 |  | 0.16 | 0.93 | 0.45 | 0.74 | 0.09 | 0.54 |
| 1 | 0.34 | 0.82 | 0.50 | 0.70 | 0.17 | 0.58 |  | 0.34 | 0.81 | 0.41 | 0.76 | 0.15 | 0.57 |
| 2 | 0.54 | 0.70 | 0.49 | 0.75 | 0.25 | 0.62 |  | 0.56 | 0.69 | 0.41 | 0.80 | **0.25** | **0.62** |
| 3 | 0.70 | 0.57 | 0.46 | 0.78 | **0.27** | **0.63** |  | 0.69 | 0.54 | 0.37 | 0.82 | 0.23 | 0.61 |
| 4 | 0.81 | 0.44 | 0.43 | 0.81 | 0.25 | 0.62 |  | 0.80 | 0.41 | 0.34 | 0.84 | 0.21 | 0.61 |
| 5 | 0.87 | 0.33 | 0.41 | 0.83 | 0.20 | 0.60 |  | 0.86 | 0.30 | 0.32 | 0.85 | 0.16 | 0.58 |
| 6 | 0.93 | 0.22 | 0.38 | 0.84 | 0.14 | 0.57 |  | 0.90 | 0.20 | 0.30 | 0.84 | 0.10 | 0.55 |
| 7 | 0.97 | 0.13 | 0.37 | 0.89 | 0.10 | 0.55 |  | 0.95 | 0.12 | 0.29 | 0.86 | 0.07 | 0.53 |
| 8 | 0.99 | 0.06 | 0.36 | 0.90 | 0.05 | 0.52 |  | 0.97 | 0.05 | 0.28 | 0.83 | 0.03 | 0.51 |
| 9 | 0.99 | 0.02 | 0.35 | 0.82 | 0.01 | 0.51 |  | 0.99 | 0.02 | 0.28 | 0.82 | 0.01 | 0.50 |
| 10 | 1.00 | 0.00 | 0.35 | **-** | 0.00 | **-** |  | 1.00 | 0.00 | 0.28 | **-** | 0.00 | - |

^a^ A higher Urgency NRS score represents more severe urgency.

^b^ Mayo endoscopic subscore 0 or 1 (excluding friability).

^c^ Geboes histologic score of 2b (absence of neutrophils in the epithelium and lamina propria; no crypt destruction, erosion, or ulceration).

^d^ Sensitivity + specificity – 1. Bold: highest value of Youden’s index. A higher value of Youden’s index indicates a better balance of sensitivity and specificity of the potential NRS score threshold in identifying participants with UC remission based on the anchor (endoscopic or histologic remission).

^e^ AUROC was calculated from a logistic regression model with the anchor variable as the dependent variable and urgency remission status (as defined by the Urgency NRS threshold) as the independent variable. Bold: highest value of AUROC.

AUROC, area under the receiver operating characteristic curve; NRS, Numeric Rating Scale; UC, ulcerative colitis.
